# ﻿Spotlighting Darwin wasps (Hymenoptera, Ichneumonidae) in Zambia: a new species and the urgent need for further exploration

**DOI:** 10.3897/zookeys.1234.144751

**Published:** 2025-04-22

**Authors:** Noah Meier, Alexandra Viertler, Meekness Kapaale, Cyprian Katongo, Tamara Spasojevic

**Affiliations:** 1 Natural History Museum Basel, Augustinergasse 2, 4051 Basel, Switzerland Natural History Museum Basel Basel Switzerland; 2 University of Bern, Institute of Ecology and Evolution, Baltzerstrasse 6, 3012 Bern, Switzerland University of Bern Bern Switzerland; 3 Department of Biosciences and Biotechnology, University of Zambia, Great East Road Campus, Lusaka, Zambia University of Zambia Lusaka Zambia; 4 Natural History Museum Vienna, Burgring 7, 1010 Wien, Austria Natural History Museum Vienna Vienna Austria

**Keywords:** Afrotropical region, biodiversity, Lake Tanganyika, parasitoids, *Pristomerus*, taxonomy

## Abstract

The parasitoid Darwin wasps (Ichneumonidae) are one of the most species-rich families of insects, with a crucial role in ecosystem functioning while many species are known as potential biological control agents. However, the group is poorly studied, especially in the Afrotropical realm, where for several countries only a handful of species have been recorded. Zambia is one of the countries with the fewest records for Darwin wasps with only 26 species reported in the largest Ichneumonidae database, “Taxapad”, from 2016 and subsequent publications. In this study, the species of Darwin wasps recorded from Zambia were reviewed and complemented with newly collected species in the Northern Province, to provide a first preliminary checklist of Darwin wasps in Zambia. Our findings increased the number of species known for Zambia to 44, which might still represent as little as 1.7% of the true diversity of the group. Despite the limited scale of the study, one new species of Afrotropical Cremastinae, *Pristomerusroussei* Meier, Viertler & Spasojevic, **sp. nov.**, is described. The study thus highlights both the substantial potential for discovery of new taxa and significant gaps in our knowledge about the Darwin wasp diversity in Zambia. To tackle these shortcomings, comprehensive collecting efforts considering the various ecotypes found in Zambia are recommended, as well as studies of natural history collections, collaborative effort by taxonomic experts, and enhancing local capacities for taxonomic research by involving students and enlarging local natural history collections.

## ﻿Introduction

Darwin wasps (Ichneumonidae) are parasitoids of holometabolous insects and spiders, playing a crucial role in natural ecosystems and as biological control agents in agriculture (Mates et al. 2012). Despite their ecological importance, the hyperdiverse Darwin wasps remain one of the most understudied insect families, with more than 25,000 species formally described (Yu et al. 2016) and as many as 60,000 species estimated worldwide (Townes 1969). The vast disparity between known and estimated species richness is particularly pronounced in certain biogeographical regions.

One of the largest gaps in species documentation is found within the Afrotropical realm, where only 2,322 species of Darwin wasps have been recorded but 9,200–15,500 species are currently expected (Townes 1969; Meier et al. 2024). Notably, the descriptions of the majority of Afrotropical species date back to the early and mid-20^th^ century, with half of the species described by a small group of entomologists, including G. Heinrich, A. Seyrig, P. Benoit, and C. Morley (Morley 1912; Benoit 1951b, 1952, 1953d, 1953c, 1953b, 1953a, 1955, 1956, 1957; Heinrich 1967a, 1967b, 1968a, 1968b, 1968c). Spatial focus of these early works was on the Democratic Republic of Congo (Benoit 1951a, 1953c, 1953d), and Madagascar (Seyrig 1932, 1934, 1952), while taxonomically only Ichneumoninae (Heinrich 1967b, 1967a, 1968b, 1968a, 1968c), and Ophioninae (Gauld and Mitchell 1978) have been more comprehensively treated. And although some countries, such as the Republic of South Africa (Rousse and van Noort 2013, 2014a; Reynolds Berry and van Noort 2016; Rousse et al. 2016), Gabon (van Noort 2004), Namibia (van Noort et al. 2000), Uganda (Hopkins et al. 2018, 2019), Central African Republic (Rousse and van Noort 2014a; Azevedo et al. 2015), and Tanzania (Russel-Smith et al. 1999) have been later systematically sampled, less than a quarter of their actual species richness of Darwin wasps seems to be recorded up to now (Meier et al. 2024). Several other Afrotropical countries have barely any faunistic records of Darwin wasps. For example, Zambia has only 26 recorded species according to the “Catalogue of world Ichneumonidae” (Yu and Horstmann 1997; Yu et al. 2016), implying a vast, untapped biodiversity awaiting exploration and formal description.

There has never been any Darwin wasp research focused on Zambia and most of the available records are simply a byproduct of studies with broader taxonomic and geographic focus. The two main catalogues dealing with Afrotropical ichneumonids, the first a catalogue of Ethiopian (= Afrotropical) Ichneumonidae by Townes and Townes (1973) and the second a revision of the subfamily Ophioninae by Gauld and Mitchell (1978), the latter updated by Rousse and van Noort (2014b), listed in sum only 13 species of ichneumonids for Zambia. Besides a few additional records from the 20^th^ Century, the record of Darwin wasps in Zambia is supplemented by recent revisionary works on Darwin wasp genera in the Afrotropical region and by a few faunistic studies (e.g., Rousse and van Noort 2015; Riedel 2016; Khalaim 2019; Giovanni and Varga 2021). Finally, with the present day studies about diversity and distribution of insects in Zambia being skewed towards economically important species, such as edible caterpillars (Kusia et al. 2023), agricultural pests (Sohati et al. 2001; Durocher-Granger et al. 2021), pollinators (Mayes and Petrillo 2017), and disease vectors (Lobo et al. 2015; Nyirenda et al. 2020; Kallu et al. 2023), there are some new species records for Darwin wasps, and parasitoids in general, coming from studies focusing on biological control (Midingoyi et al. 2016; Durocher-Granger et al. 2021). While such studies are beneficial, they alone cannot provide a comprehensive species list of Zambian Darwin wasps and a map of their distribution.

As a fundamental step towards a more complete checklist of Darwin wasps in Zambia, we provide here a first preliminary checklist, which is based on thorough literature research and a small-scale collecting effort in the Northern Province of Zambia. The goal of this preliminary checklist is to record the status quo of our knowledge about the Zambian fauna, provide a basis for future research, and highlight the gap between the known and expected species richness in Zambia.

## ﻿Materials and methods

### ﻿Abbreviations of depositories

**NHMUK**Natural History Museum, London, UK (Gavin Broad)

**CABI**CAB International, Delémont, Switzerland (Marc Kenis)

**LKG** Oberösterreichische Landes-Kultur GmbH, Linz (Esther Ockermüller)

**LMNH**Livingstone Museum, Zambia (Martha Imakando)

**MNHN**Muséum national d’Histoire naturelle, Paris, France (Claire Villemant)

**MZH**Finnish Museum of Natural History, Helsinki (Juho Paukkunen)

**NHMZ** Natural History Museum of Zimbabwe

**NMBS**Naturhistorisches Museum (Seraina Klopfstein)

**RMNH**Naturalis, Leiden, The Netherlands (Frederique Bakker)

**SAMC**Iziko South African Museum, Cape Town, South Africa (Simon van Noort)

**TC** Townes Collection, Ann Arbor, Michigan, USA (now in Logan, Utah) (David Wahl)

**UNZA**University of Zambia, Lusaka, Natural history collection (Philip Nkunika)

**ZSM** Zoologische Staatssammlung München

### ﻿Sampling locality and material

Zambia is a landlocked country located in the Sub-Equatorial Afrotropics (Burkart 2023). It covers an area of 752,612 square kilometres and lies between latitudes 8° and 18° south and longitudes 22° and 34° east. Based on the amount of rainfall received but also to a limited extent according to soils and other climatic characteristics, Zambia is divided into three agro-ecological regions (Zones I, II, and III). Our study site belongs to the agro-ecological Zone III, which covers northern and north-western parts of the country, and it is classified as a high rainfall region, receiving more than 1000 mm rainfall per annum on average (Dautu et al. 2012). Based on the dominant vegetation type, our study sites belong to the Central Zambezian Miombo woodlands ecoregion, which covers circa 50% of Zambian territory (Malambo and Syampungani 2008). This ecoregion is characterised by a long dry season, up to seven months, and a rainy season from approximately November to March. It is a part of the Zambezian regional centre of endemism, and it is home to dozens of endemic plant species.

**Figure 1.**
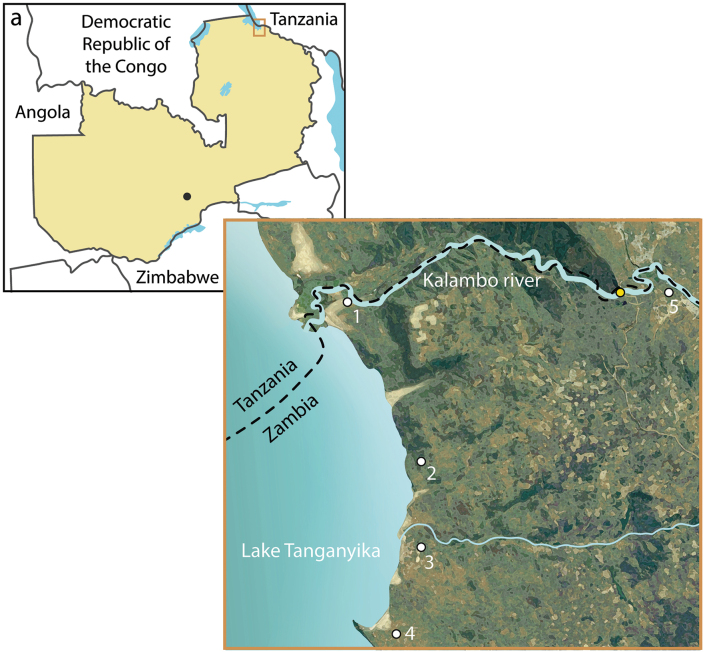
Maps of the sampling locality **a** map of Zambia, where Lusaka (black dot) and our sampling area (orange rectangle) are marked **b** sampling sites: **1** Kalambo River delta **2** Kalambo falls lodge **3** Chitili, with a nearby stream **4** Isanga Bay lodge **5** Kalambo River above Kalambo waterfall (yellow dot).

We collected specimens in the Northern Province of Zambia in the region of Lake Tanganyika around Mpulungu for two weeks, from the end of August to the beginning of September 2023 (Fig. 1). This fieldwork was conducted at the end of the dry season, following a couple of months without rain. Most of the sampling occurred in well-watered areas, such as those near lodges, streams, and the shores of Lake Tanganyika. Specimens were collected using sweep nets at all collection sites, and at Kalambo falls lodge additionally with Malaise traps, pitfall traps and light traps. All samples were stored in 80% ethanol on site and subsequently sorted and dry-mounted at the NMBS in Switzerland. Collected ichneumonids are currently at the NMBS but they will be divided between the NMBS and LMNH, where ichneumonid holotypes will be deposited in the NMBS, and paratypes in the LMNH and NMBS. For this study, we obtained all necessary collection and export permits, issued by the National Health Research Authority (NHRA) of Zambia. The permits are in accordance with the principles and regulations of the Nagoya protocol as confirmed by the Ministry of Lands and Natural Resources in Zambia.

### ﻿Species descriptions

Morphological terminology follows Broad et al. (2018). The dimensions of the face (excluding the clypeus) are measured from the antennal sockets to the tentorial pits and from the inner margin of one eye to the inner margin of the other eye at level of the antennal sockets. The dimensions of the clypeus are measured between the mandibular bases and from the apical margin of clypeus to the height of the tentorial pits (largest distance). Images of the collected samples were taken with a Keyence VHX-6000 using stacking and stitching techniques.

### ﻿Lab work

We sequenced the barcoding portion of cytochrome oxidase subunit 1 (COI) gene as a reference for a newly described species. One leg with coxa from a female and a male paratype was used to extract DNA. DNA extracts and the voucher specimens are stored at the NMBS. Extractions were done with the DNeasy blood and tissue kit from Qiagen according to the standard protocol, but with a prolonged digestion step over night at 56 °C and two elusion steps with only 50 µl each. We used previously published primers for COI (Folmer et al. 1994) for PCR with annealing temperatures of 50 °C. The quality of the PCR products was then checked on a 2% agarose electrophoresis gel and successful amplification were sent for cleanup and sequencing to Macrogen Europe in the Netherlands. Sequence editing was done with the CodonCode Aligner software version 9.0.1.3 (CodonCode Corporation, Dedham, MA, USA), while sequence alignment was done with MEGAX (Kumar et al. 2018) using the MUSCLE alignment algorithm with default settings. No gaps or stop codons were detected. Uncorrected p-distances for COI were calculated in MEGAX, using pairwise deletions (Kumar et al. 2018). The newly generated sequences are deposited in GenBank, with accession numbers provided in the respective species descriptions.

## ﻿Results

### ﻿Species description

We here describe one Cremastinae species new to science.

#### ﻿*Pristomerus* Curtis, 1836

##### ﻿ Pristomerus roussei

Taxon classificationAnimaliaHymenopteraIchneumonidae

Meier, Viertler & Spasojevic sp. nov.

2245CE68-BC4D-5E6E-8076-0E7ED59027E7

https://zoobank.org/615E6352-F991-4C94-AD8B-DCB9A85D0B5E

Fig. 2

###### Material examined.

***Holotype*.** • 1 ♀, ZM **Northern Province**, Mbala, Kalambo Falls (above waterfall), sweep net, 1170 m, -8.5961/31.2478, 21.viii–2.ix.2023, leg. N. Meier, T. Spasojevic, A. Viertler (NMBS). ***Paratypes*.** • 1 ♂, ZM **Northern Province**, Mbala, Kalambo Falls (above waterfall), sweep net, 1170 m, -8.5961/31.2478, 21.viii–2.ix.2023, leg. N. Meier, T. Spasojevic, A. Viertler (NMBS). • 1 ♂, ZM Mpulungu, Chitili stream, sweep net, -8.6390/31.2035, 21.viii–2.ix.2023, leg. N. Meier, T. Spasojevic, A. Viertler, voucher: 20-538 (NMBS). • 2 ♂, ZM Mpulungu, Kalambo delta, sweep net, 777 m, -8.5964, 31.1844, 21.viii–2.ix.2023, leg. N. Meier, T. Spasojevic, A. Viertler (LMNH). • 1 ♀, ZM Mpulungu, Mpulungu town, sweep net, 777 m, -8.7621, 31.1138, 21.viii–2.ix.2023, leg. N. Meier, T. Spasojevic, A. Viertler, voucher: 20-537 (NMBS). • 1 ♀, ZM Mpulungu, Kalambo Falls Lodge, sweep net, 786 m, -8.6241/31.2011, 21.viii–2.ix.2023, leg. N. Meier, T. Spasojevic, A. Viertler (LMNH). • 1 ♀, ZM **Northern Western Province**, 150 km W Solwezi, Ntambu, 12°18'S, 25°10'E; 11.11.2005; leg. M. Halada (LKG).

**Figure 2.**
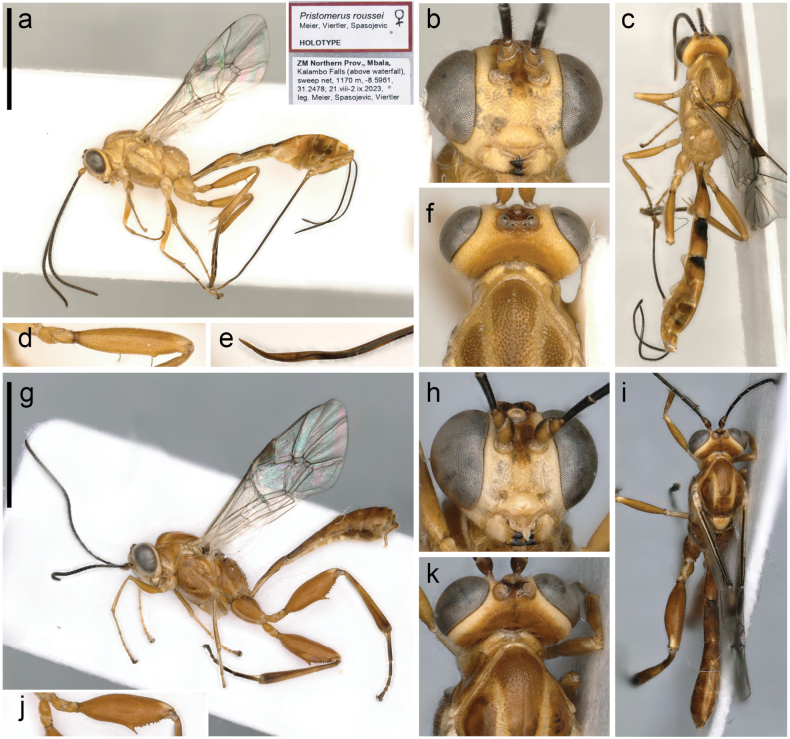
*Pristomerusroussei* sp. nov. Meier, Viertler & Spasojevic. **a–f** holotype (female) **a** habitus, lateral view, with labels **b** face, frontal view **c** habitus, dorsal view **d** hind femur **e** ovipositor tip **f** mesoscutum and occiput, dorsal view. **g–k** paratype (male, with the same collection data as the holotype) **g** habitus, lateral view **h** face, frontal view **i** habitus, dorsal view **j** hind femur **k** mesoscutum and occiput dorsal view. Scale bars: 3 mm.

###### Diagnosis.

Moderate size; pale yellow with black spots on tergites 1–3 antero-dorsally; femora apically white-dotted; pterostigma anteriorly white; occiput without dark spots; face densely and very shallowly punctate; clypeus transverse with dispersed punctures dorsally and almost smooth ventrally; remainder of head coriaceous; malar space shorter than base of mandible; antenna with 30–33 flagellomeres, penultimate flagellomere quadrate; distance between posterior ocelli approx. as wide as one posterior ocellus; mesosoma densely punctate with pronotum and speculum nearly smooth; female femoral tooth distinct but clearly wider than high, followed by a row of minute denticles; ovipositor moderately long, apically weakly sinuous. Male with ocelli enlarged, inner margins of eyes slightly diverging ventrally, femur and femoral tooth stouter with femoral tooth long and acute, area superomedia much more slender than in female.

###### Differential diagnosis.

*Pristomerusroussei* sp. nov. is morphologically very close to *Pristomerusmasai* Rousse & van Noort, 2015, which is suspected to represent a species-complex (Rousse and van Noort 2015). In the dichotomous key of Rousse and van Noort (2015), *P.roussei* ends at couplet #36. It can be differentiated from *P.masai* by a wider distance between the posterior ocelli, a quadrate penultimate flagellomere (females and males) and the lack of dark marks on the occiput (females). *Pristomerusroussei* can be differentiated from *P.wolof* Rousse & van Noort, 2015 by the more strongly developed femoral tooth (females and males), the quadrate penultimate flagellomere rather than elongate (females and males), and by its more extensive dark metasomal colour (females).

###### Description.

**Female**: 4 specimens (measurements of the holotype in brackets). ***Colour*.** Pale yellow. Scape, pedicel, frons, stemmaticum and occiput pale brown; mandibular teeth and flagellum black; mesoscutum apart from notauli and posterior parts orange; wings hyaline, venation brown, pterostigma black, proximal corner translucent; legs orange, femur apically with a white spot, hind tibia basally and apically slightly infuscate, hind tarsus except basal 0.2 of first tarsal segment black; tergites 1–3 on the anterior half black; ovipositor sheath black; ovipositor dark brown, basally and apically testaceous.

***Head*.** Face 2.5× (2.5×) wider than high, densely and very shallowly punctate; inner margins of eyes subparallel, distance between posterior ocelli ~ 1.0× (1.0×) as wide as diameter of one posterior ocellus, distance between posterior ocelli and margin of compound eye 1.1× (1.1×) wider than diameter of one posterior ocellus; clypeus 2.0× (2.0×) wider than high and moderately convex, with dispersed punctures dorsally, almost smooth ventrally; malar space ~ 0.7–0.75× (0.75×) base of mandible; frons, vertex and temple coriaceous; occipital carina joining hypostomal carina distinctly above mandible base; antenna with 31–33 (33) flagellomeres, penultimate flagellomere ~ 1.0× (1.0×) as long as wide.

***Mesosoma*.** Moderately elongate; pronotum nearly smooth posteriorly, with longitudinal striations in its impression and punctures ventrally; mesopleuron and metapleuron with space between punctures less than maximum diameter of punctures, between punctures smooth and shining, mesopleuron with a shallow oblique and transversely strigose furrow below speculum, speculum smooth; mesoscutum densely punctate, between punctures smooth and shiny, notaulus distinct on anterior third of mesoscutum; scutellum densely punctate; propodeum densely punctate, propodeal carination complete, superomedial area ~ 2.3–2.5× (2.3×) as long as its maximum width.

***Wings*.** Fore wing ~ 4.5–4.9 mm (4.9 mm) with M between 2rs-m and 2m-cu ~ 1.25–1.5× (1.25×) as long as 2rs-m, 2m-cu with a small bulla covering anterior 0.2–0.4 of 2m-cu; hind wing with nervellus intercepted at lower 0.7 (0.7), second abscissa of CU not pigmented.

***Legs*.** Tooth on hind femur distinct, clearly wider than high, followed by a row of minute denticles. Hind tibia with dispersed spines between normal setation. Tarsal claws pectinate with two distinct denticles.

***Metasoma*.** Posterior half of tergite 1, tergite 2, and anterior of tergite 3 longitudinally aciculate, following tergites coriaceous; thyridium elongate, elliptic; ovipositor sheath ~ 1.5× as long as hind tibia, apically weakly to moderately sinuous; ovipositor notch rather weak, its distance from ovipositor tip ~ 1.5× basal width of first tarsal segment of hind leg.

**Male**: 4 specimens. Similar to female; stemmaticum with ocelli slightly raised and ocelli enlarged, distance between posterior ocelli approx. as wide as diameter of one posterior ocellus, distance between posterior ocelli and margin of compound eye less than half diameter of one posterior ocellus; inner margins of eyes distinctly diverging ventrally; antenna with 29–31 flagellomeres; mesoscutum laterally almost smooth, impunctate; femur swollen, femoral tooth stronger, long and acute, apically with a distinct row of denticles; dark marks on metasoma sometimes absent or reduced.

###### Etymology.

Dedicated to Pascal Rousse, who contributed greatly to our current understanding of Afrotropical *Pristomerus* species.

### ﻿Molecular data

Sequences of the COI barcode for the female and male paratype are given below. The pairwise distance (p-distance) between these two sequences is 0.3%. The best match according to the NCBI standard nucleotide BLAST search belongs to the Afrotropical *Pristomeruspallidus* (Kriechbaumer, 1884) (GenBank: MF673618.1) with a p-distance of 5.6–6.0%.

GenBank Accession ID: PV176400, NMBS:20-537, female paratype; 645 bp.

ATTTTTGGTATATGATCTGGGATAATTGGATCTTCTATAAGATTAATTATTCGATTAGAATTAGGGAATCCGGGGTCTTTAATTAATAATGATCAAATTTATAATTCTATAATTACAATACATGCTTTTATTATAATTTTTTTTATAGTTATACCAGTTATAATTGGAGGGTTTGGAAATTGATTAATTCCTCTAATATTAGGAGCTCCAGATATAGCTTTTCCTCGAATAAATAATTTAAGATTTTGATTATTAATTCCTTCGTTAATGATATTAATTATGAGATCAATTACTAATCAAGGAGTGGGTACAGGATGAACAATATATCCTCCTTTATCATTAAATTTAAATCAAGAAGGTATATCAATAGATTTATCTATTTTTTCTTTACATTTAGCAGGTATATCTTCAATTTTAGGATCTATTAATTTTATTTCTACTATTATAAATATAAAAATTTTTGATTCAAAATTAGATCAATTAACTTTATTTTCTTGATCAATTAATATTACTACAATTTTATTATTATTAGCTGTTCCAGTATTAGCAGGAGCAATTACTATAATTTTAACAGATCGAAATTTAAATACTTCTTTTTTTGATCCAAGTGGAGGAGGAGATCCAATTTTATTTCAACATTTATTT.

GenBank Accession ID: PV176401, NMBS:20-538, male paratype; 645 bp.

ATTTTTGGTATATGATCTGGGATAATTGGATCTTCTATAAGATTAATTATTCGATTAGAATTAGGGAATCCGGGGTCTTTAATTAATAATGATCAAATTTATAATTCTATAATTACAATACATGCTTTTATTATAATTTTTTTTATAGTTATACCAGTTATAATTGGAGGGTTTGGAAATTGATTAATTCCTCTAATATTAGGAGCTCCAGATATAGCTTTTCCTCGAATAAATAATTTAAGATTTTGATTATTAATTCCTTCGTTAATAATATTAATTATGAGATCAATTACTAATCAAGGAGTGGGTACAGGATGAACAATATATCCTCCTTTATCATTAAATTTAAATCAAGAAGGTATATCAATAGATTTATCTATTTTTTCTTTACATTTAGCAGGTATATCTTCAATTTTAGGATCTATTAATTTTATTTCTACTATTATAAATATAAAAATTTTTGATTCAAAATTAGATCAATTAACTTTATTTTCTTGATCAATTAATATTACTACAATTTTATTATTATTAGCTGTTCCAGTATTAGCAGGAGCAATTACTATAATTTTAACAGATCGAAATTTAAATACTTCTTTTTTTGATCCAAGTGGGGGAGGAGATCCAATTTTATTTCAACATTTATTT.

### ﻿Preliminary checklist

By combining published species records from literature and the new material collected in this study, we provide a first preliminary checklist for Darwin wasps in Zambia with 44 species (Table 1). Except for the cosmopolitan *Diplazonlaetatorius* (Fabricius, 1781) and widely distributed *Xanthopimplaromani* Krieger, 1915, all other newly collected and identified species (*n* = 14) were previously unrecorded for Zambia (*). We also found literature records of four additional species in Zambia (#) that were not included in the Yu and Horstmann (1997) catalogue nor its electronic version (Yu et al. 2016). Moreover, the subfamilies Anomaloninae, Banchinae, Orthocentrinae, and Phygadeuontinae and 17 genera (*Anomalon*, *Spilopimpla*, *Syzeuctus*, *Venturia*, *Porizon*, *Xanthocampoplex*, *Temelucha*, *Trathala*, *Cryptus*, *Goryphus*, *Stenarella*, *Osprynchotus*, *Afromevesia*, *Triclistus*, *Megastylus*, *Paraphylax*, *Theronia*) are recorded from Zambia for the first time (*). Outstandingly, the record of the genus *Porizon* is also the first for the Afrotropical realm. In the list of records below, verbatim data are given in square brackets.

**Table 1.** Preliminary checklist of species recorded for Zambia based on literature records and our field study in the Northern Province of Zambia, Mpulungu district. In total, 44 species are recorded, one of which is described as new to science in this publication. * Previously unrecorded species; # missing from Yu and Horstmann (1997, 2016) catalogue; [?] no repository mentioned in the reference.

Subfamily	Species	Literature	Our study	Repository	Reference
Brachycyrtinae	*Brachycyrtuslucchii* Di Giovanni & Varga, 2021	x		NHMUK	Di Giovanni and Varga (2021)
Campopleginae	*Diadegmamollipla* (Holmgren, 1868)	x		[?]	Cruickshank and Ahmad (1973)
	*Charopselectrinus Vas, 2020		x	NMBS, LMNH	
	*Venturiaaquila Vas, 2019		x	NMBS	
	*Xanthocampoplexoneili (Cameron, 1905)		x	NMBS	
Cremastinae	*Pristomerusbemba* Rousse & van Noort, 2015	x		SAMC	Rousse and van Noort (2015)
	*Pristomerusbullis* Fitton in Polaszek et al. 1994	x		SAMC	Rousse and van Noort (2015)
	*Pristomerusroussei sp. nov.		x	NMBS, LMNH	
	*Temeluchabasiornata (Cameron, 1911)		x	NMBS, LMNH	
	*Trathalaannulicornis (Tosquinet, 1896)		x	NMBS, LMNH	
Cryptinae	*Coccygodessubquadratus* (Waterston, 1927)	x		[?]	Benoit (1951a); Waterston (1927)
	*Osprynchotusgigas Kriechbaumer, 1894		x	NMBS, LMNH	
	*Zonocryptusformosus* (Brullé, 1846)	x		[?]	Waterston (1927)
Diplazontinae	*Diplazonlaetatorius* (Fabricius, 1781)	x	x	NMBS	
Ichneumoninae	#*Ctenocharesrufithorax* (Kriechbaumer, 1894)	x		NHMUK	Morley (1915)
	#*Ischnojoppaluteator* (Fabricius, 1798)	x		NHMUK	Morley (1915)
	#*Leptophatnuscrococephalusrubricaput* (Morley, 1919)	x		ZSM	Heinrich (1967)
Metopiinae	*Metopiusalbipictus* Tosquinet, 1896	x		LKG	Riedel (2016)
	*Metopiusclathratus* Benoit, 1965	x		NHMUK	Riedel (2016)
	*Metopiusdiscolor* Tosquinet, 1896	x		NHMUK	Durocher-Granger et al. (2021); Riedel (2016); Townes and Townes (1973)
	*Metopiusrufigasterzambiensis* Riedel, 2016	x		LKG	Riedel (2016)
	*Metopiuszuluanus* Benoit, 1965	x		LKG	Riedel (2016)
Ophioninae	*Enicospilusalbiger* (Kriechbaumer, 1894)	x		SAMC	Rousse and van Noort (2014b)
	*Enicospilusantefurcalis* (Szépligeti, 1908)	x		NHMUK	Gauld and Mitchell (1978)
	*Enicospilusbiimpressus* (Brullé, 1846)	x		NHMUK	Gauld and Mitchell (1978)
	*Enicospiluscapensis* (Thunberg, 1822)	x		CABI	Durocher-Granger et al. (2021)
	*Enicospilusfenestralis* (Szépligeti, 1906)	x		NHMUK	Gauld and Mitchell (1978)
	*Enicospilushelvolus* Gauld & Mitchell, 1978	x		TC	Gauld and Mitchell (1978)
	*Enicospiluslaquaetus* (Enderlein, 1921)	x		NHMUK	Gauld (1982)
	*Enicospilusmauritii* (Saussure, 1892)	x		NHMUK	Morley (1912)
	*Enicospilusnefarius* Gauld & Mitchell, 1978	x		TC	Gauld and Mitchell (1978)
	*Enicospilusnops* Gauld & Mitchell, 1978	x		NHMUK	Gauld and Mitchell (1978)
	#*Enicospilustransvaalensis* Cameron, 1911	x		TC	Gauld and Mitchell (1978)
	*Enicospiluswatshami* Gauld, 1982	x		NHMUK	Gauld (1982)
	*Euryophionlatipennis* (Kirby, 1896)	x		SAMC	Rousse and van Noort (2014b)
	*Euryophionnigripennis Cameron, 1906		x	NMBS	
	*Lepiscelusdistans* (Seyrig, 1935)	x		TC	Gauld and Mitchell (1978)
Pimplinae	*Theronialurida Tosquinet, 1896		x	NMBS	
	*Xanthopimplaromani* Krieger, 1915	x	x	NHMZM, NMBS, LMNH	Benoit (1953b)
	*Xanthopimplastemmator* (Thunberg, 1822)	x		[?]	Midingoyi et al. (2016)
Tersilochinae	*Diaparsisinterstitialis* Khalaim, 2013	x		MZH	Khalaim (2019)
	*Diaparsismostovskii* Khalaim, 2013	x		RMNH	Khalaim (2019)
	*Diaparsisvoluptuosa* Khalaim, 2013	x		MZH	Khalaim (2019)
Tryphoninae	*Zambionmonodon* Kasparyan, 1993	x		MZH	Kasparyan (1993)

#### ﻿Anomaloninae

*Anomaloncf.flavomaculatum (Cameron,1905)

**Records.** 2 ♂♂, ZM Northern Province, Mpulungu, Mbala, Kalambo falls, (above waterfall), 1170 m, -8.5961/31.2478, 29.viii.2023, leg. N. Meier, T. Spasojevic, A. Viertler (NMBS).

**Remarks.** There are five additional *Anomalon* specimens, probably belonging to two or three new species, which are currently being treated in the genus revision of Heinz Schnee (H. Schnee, pers. comm.).

#### ﻿Banchinae

* **Spilopimplacf.chappuisi (Seyrig, 1935)**

**Records.** 1 ♀, ZM Northern Province, Mpulungu, Kalambo River Delta, sweep-net, -8.5965/31.1844, 21.viii–2.ix.2023, leg. N. Meier, T. Spasojevic, A. Viertler (NMBS).

* ***Syzeuctus* sp.**

**Records.** 1 ♀, ZM Northern Province, Mpulungu, Kalambo River Delta, sweep-net, -8.5965/31.1844, 21.viii–2.ix.2023, leg. N. Meier, T. Spasojevic, A. Viertler (NMBS).

#### ﻿Brachycyrtinae

***Brachycyrtuslucchii* Di Giovanni & Varga, 2021**

**Records.** 1 ♀, ZM Lusaka Province, 15 km E. Lusaka, 4–15.xii.1979, R. A. Beaver (NHMUK).

1 ♂, ZM Lusaka Province, 15 km E. Lusaka, 11–21.i.1980, R. A. Beaver (NHMUK).

1 ♂, ZM Lusaka Province, Lusaka, 1–14.iv.1980, R. A. Beaver (NHMUK) (Di Giovanni and Varga 2021).

#### ﻿Campopleginae

***Diadegmamollipla* (Holmgren, 1868)**

**Records.** Unspecified, ZM (Cruickshank and Ahmad 1973).

* ***Charopselectrinus* Vas, 2020**

**Records.** 1 ♀, ZM Northern Province, Mpulungu, Kalambo Falls Lodge, sweep-net, -8.6241/31.2011, 21.viii–02.ix.2023, leg. N. Meier, T. Spasojevic, A. Viertler (NMBS, LMNH). 1 ♀ same as previous except collected by a Malaise trap.

***Charops*** sp.

**Records.** 3 ♀/♂, ZM Southern Province, Kuzungula district (Chipabika et al. 2023).

**Remarks.** These specimens were not identified to species level in the original publication. Future examination of the specimens is needed to clarify whether these belong to *C.electrinus* or another *Charops* species.

* ***Venturiaaquila* Vas, 2019**

**Records.** 1 ♀, ZM Northern Province, Mpulungu, Kalambo Falls Lodge, light trap, -8.6241/31.2011, 21.viii–2.ix.2023, leg. N. Meier, T. Spasojevic, A. Viertler (NMBS).

**Remarks.** The species is morphologically very similar to the slightly smaller *Venturiadesertorum* Horstmann, 2008 from southern Algeria (Horstmann 2008). Future studies should include larger series and molecular data to test whether these species are distinct or rather variations.

* ***Porizon* sp.**

**Records.** 1 ♂, ZM Northern Province, Mpulungu, Kalambo Falls Lodge, sweep net, -8.6241/31.2011, 21.viii–02.ix.2023, leg. N. Meier, T. Spasojevic, A. Viertler (NMBS).

**Remarks.** This is the first record of this genus for the Afrotropical region.

* ***Xanthocampoplexoneili* (Cameron, 1905)**

**Records.** 1 ♂, ZM Northern Province, Mpulungu, Kalambo Falls Lodge, -8.6241/31.2011, 21.viii–02.ix.2023, leg. N. Meier, T. Spasojevic, A. Viertler (NMBS).

#### ﻿Cremastinae

***Pristomerusbemba* Rousse & van Noort, 2015**

**Records.** 1 ♀, ZM Eastern Province, South Luangwa, nr Mfuwe ca 10 km E. Mfuwe Malinba village vicinities, 12.XII.2011, Gumovsky leg. SAM–HYM–P047391” (holotype, SAMC) (Rousse and van Noort 2015).

***Pristomerusbullis* Fitton in Polaszek et al. 1994**

**Records.** 1 ♂ ZM Eastern Province, South Luangwa, near Mfuwe sweeping on the dried egg tree 09.XII.2011 Gumovsky; Mopane tree [Colophospermum mopane, Fabaceae] SAM–HYM–P049439” (SAMC) (Rousse and van Noort 2015).

*Pristomerusroussei sp. nov.

**Records.** 1 ♀, ZM Northern Province, Mbala, Kalambo Falls (above waterfall), sweep net, 1170 m, -8.5961/31.2478, 21.viii–2.ix.2023, leg. N. Meier, T. Spasojevic, A. Viertler (holotype, NMBS).

1 ♂, ZM Northern Province, Mbala, Kalambo Falls (above waterfall), sweep net, 1170 m, -8.5961/31.2478, 21.viii–2.ix.2023, leg. N. Meier, T. Spasojevic, A. Viertler (paratype, NMBS).

1 ♂, “ZM Northern Province, Mpulungu, Chitili stream, sweep net, -8.6390/31.2035, 21.viii–2.ix.2023, leg. N. Meier, T. Spasojevic, A. Viertler, voucher: 20-538 (paratype, NMBS).

2 ♂♂, “ZM Northern Province, Mpulungu, Kalambo delta, sweep net, 777 m, -8.5964, 31.1844, 21.viii–2.ix.2023, leg. N. Meier, T. Spasojevic, A. Viertler (paratype, LMNH).

1 ♀, “ZM Northern Province, Mpulungu, Mpulungu town, sweep net, 777 m, -8.7621, 31.1138, 21.viii–2.ix.2023, leg. N. Meier, T. Spasojevic, A. Viertler, voucher: 20-537 (paratype, NMBS).

1 ♀, ZM Northern Province, Mpulungu, Kalambo Falls Lodge, sweep net, 786 m,-8.6241/31.2011, 21.viii–2.ix.2023, leg. N. Meier, T. Spasojevic, A. Viertler (paratype, LMNH).

1 ♀, ZM Northern Western Province, 150 km W Solwezi, Ntambu, 12°18'S, 25°10'E; 11.11.2005; leg. M. Halada (paratype, LKG).

*Temeluchabasiornata (Cameron, 1911)

**Records.** 2 ♀♀, ZM Northern Province, Mpulungu, Kalambo Falls Lodge, -8.6241/31.2011, 21.viii–02.ix.2023, leg. N. Meier, T. Spasojevic, A. Viertler (LMNH).

1 ♀ 1 ♂, ZM Northern Province, Mupulung, Tomo Sakalani, Isanga Bay Lodge, sweep-net, -8.6554/31.1947, 21.viii–2.ix.2023, leg. N. Meier, T. Spasojevic, A. Viertler (NMBS).

**Remarks.** This species is quite variable in colouration, e.g., some specimens have black marks on the vertex and the mesoscutum. It can be differentiated from the similar species *Temeluchapicta* (Holmgren, 1868) by the lack of black spots on the mesopleuron (Rousse et al. 2011).

*Trathalaannulicornis (Tosquinet, 1896)

**Records.** 1 ♀, ZM Northern Province, Mpulungu, Chitili stream, sweep net, -8.6390/31.2035, 21.viii–2.ix.2023, leg. N. Meier, T. Spasojevic, A. Viertler (NMBS).

2 ♀♀, ZM Northern Province, Mpulungu, Kalambo Falls Lodge, sweep net, -8.6241/31.2011, 21.viii–2.ix.2023, leg. N. Meier, T. Spasojevic, A. Viertler (NMBS, LMNH). 1 ♀ same as previous except collected by a Malaise trap.

**Remarks.** Currently all *Trathala* specimens with white bands on the flagellum are treated as *T.annulicornis*. However, we have seen various specimens that differ from the holotype of *T.annulicornis* in facial dimensions (from the Malagasy region, Rousse et al. 2011) or relative ovipositor length (in our material). Due to the limited number of specimens in our study, we currently refrain from describing new species within this complex.

#### ﻿Cryptinae

***Coccygodessubquadratus* (Waterston, 1927)**

**Records.** 1 ♀, ZM Lusaka Province, Chilanga, 4,000 ft, 6.x.1913 (F. V. Bruce Miller) (Waterston 1927).

Unspecified, ZM (Benoit 1951a)

*Cryptus sp.

**Records.** 1 ♂, ZM Northern Province, Mpulungu, Kalambo River Delta, sweep net, -8.6390/31.2035, 21.viii–2.ix.2023, leg. N. Meier, T. Spasojevic, A. Viertler (NMBS).

*Goryphus sp.

**Records.** 1 ♀, ZM Northern Province, Mpulungu, Kalambo Falls Lodge, sweep net, -8.6241/31.2011, 21.viii–2.ix.2023, leg. N. Meier, T. Spasojevic, A. Viertler (NMBS).

1 ♀, ZM Northern Province, Mpulungu, Chitili stream, sweep net, -8.6390/31.2035, 21.viii–2.ix.2023, leg. N. Meier, T. Spasojevic, A. Viertler (NMBS).

*Osprynchotusgigas Kriechbaumer, 1894

**Records.** 1 ♀, ZM Northern Province, Mpulungu, Kalambo Falls Lodge, sweep net, -8.6241/31.2011, 21.viii–2.ix.2023, leg. N. Meier, T. Spasojevic, A. Viertler (LMNH).

1 ♀, ZM Northern Province, Mpulungu, Kalambo Falls Lodge, sweep net, -8.6241/31.2011; 29.ix.2021, leg. F. Ronco, F. Schedel & A. Indermaur (NMBS).

6 ♂♂, ZM Northern Province, Mpulungu, Kalambo Falls Lodge, sweep net, -8.6241/31.2011, 21.viii–2.ix.2023, leg. N. Meier, T. Spasojevic, A. Viertler (NMBS).

2 ♂♂, ZM Northern Province, Mpulungu, Chitili stream, sweep net, -8.6390/31.2035, 21.viii–2.ix.2023, leg. N. Meier, T. Spasojevic, A. Viertler (LMNH).

1 ♂, ZM Northern Province, Mpulungu, Kalambo River Delta, sweep net, -8.5965/31.1844, 21.viii–2.ix.2023, leg. N. Meier, T. Spasojevic, A. Viertler (LMNH).

1 ♂, ZM Northern Province, Mbala, Kalambo Falls (above waterfall), sweep net, 1170 m, -8.5961/31.2478, 21.viii–2.ix.2023, leg. N. Meier, T. Spasojevic, A. Viertler (LMNH).

*Stenarella sp.

**Records.** 1 ♂, ZM Northern Province, Mpulungu, Kalambo Falls Lodge, sweep net, -8.6241/31.2011, 21.viii–2.ix.2023, leg. N. Meier, T. Spasojevic, A. Viertler (NMBS).

***Zonocryptusformosus* (Brullé, 1846)**

**Records.** 1 ♀, ZM Eastern Province, on road from Chipata [Fort Jameson] to Lundazi [Landazi], 4000ft, 7–14.vi.1910 (S. A. Neave) (Waterston 1927).

***Zonocryptus* sp.**

**Records.** 1 ♀, ZM Northern Province, Mpulungu, Kalambo Falls Lodge, sweep net, -8.6241/31.2011, 21.viii–2.ix.2023, leg. N. Meier, T. Spasojevic, A. Viertler (NMBS).

**Remarks.** Due to the lack of reference material, identification of two male Cryptinae was not possible to genus level.

#### ﻿Diplazontinae

***Diplazonlaetatorius* (Fabricius, 1781)**

**Records.** Unspecified, ZM Southern Province, Choma, 17.05.2006 (Klopfstein et al. 2010).

1 ♀, ZM Northern Province, Mpulungu, Kalambo Falls Lodge, sweep net, -8.6241/31.2011, 21.viii–2.ix.2023, leg. N. Meier, T. Spasojevic, A. Viertler (LMNH).

#### ﻿Ichneumoninae

#***Ctenocharesrufithorax* (Kriechbaumer, 1894)**

**Records.** Unspecified, ZM, Upper Luangwa River, viii.1910 (Neave) (NHMUK) (Morley 1915).

#***Ischnojoppaluteator* (Fabricius, 1798)**

**Records.** Unspecified, ZM, Luangwa River, 16–1800ft., September (NHMUK) (Morley 1915).

# ***Leptophatnuscrococephalusrubricaput* (Morley, 1919)**

**Records.** Unspecified, ZM Northern Province, Mbala [Abercorn] (ZSM) (Heinrich 1967b).

*Afromevesiacf.merusilvae (Heinrich, 1968)

**Records.** 1 ♀, ZM Northern Province, Mupulung, Tomo Sakalani, Isanga Bay Lodge, sweep-net, -8.6550/31.1946, 26.viii.2023, leg. N. Meier, T. Spasojevic, A. Viertler (LMNH).

1 ♀, ZM Northern Province, Mupulung, Tomo Sakalani, Isanga Bay Lodge, sweep-net, -8.6550/31.1946, 26.viii.2023, leg. N. Meier, T. Spasojevic, A. Viertler (NMBS).

**Remark.** Our specimens slightly differ from the holotype description of *Afromevesiamerusilvae* (Heinrich 1968c). Our two specimens have almost completely white trochanters (~ 90% white), while the holotype description mentions only the apical part of the trochanters white. Also, our specimens both have 27 antennal segments, while the holotype of *A.merusilvae* is described as having 30 segments.

#### ﻿Metopiinae

***Metopiusalbipictus* Tosquinet, 1896**

**Records.** 1 ♂, ZM Copperbelt Province., 25 km W Chingola 1600 m, 16.i.2006, leg. R. Kneco (LKG) (Riedel 2016).

***Metopiusclathratus* Benoit, 1965**

**Records.** 1 ♂, ZM Lusaka Province, 15 km E. Lusaka, 22–31.i.1980 R.A. Beaver (NHMUK) (Riedel 2016).

***Metopiusdiscolor* Tosquinet, 1896**

**Records.** Unspecified, ZM (Townes and Townes 1973).

1 ♀, ZM Lusaka Province, Lusaka, iv.1980, leg. R.A. Beaver (NHMUK) (Riedel 2016). 2 ♀♀, ZM Lusaka; IV.1980, leg. R.A. Beaver (NHMUK) (Riedel 2016).

Unspecified, Central Province, Chisamba, Golden Valley Agricultural Research Trust, (14,967,373; 28,097,464, altitude: 1147 m) (Durocher-Granger et al. 2021).

***Metopiusrufigasterzambiensis* Riedel, 2016**

**Records.** 1 ♂, holotype, ZM Copperbelt Province, 45 km SE Kitwe, 12–15.i.2003, leg. J. Halada (LKG), (Riedel 2016).

***Metopiuszuluanus* Benoit, 1965**

**Records.** 5 ♂♂, ZM Copperbelt Province, 25 km W Chingola 1600 m, 16.i.2006, leg. R. Kmeco (LKG) (Riedel 2016).

*Triclistus sp.

**Records.** 1 ♂, ZM Northern Province, Mpulungu, Kalambo Falls Lodge, light trap, -8.6241/31.2011, 21.viii–2.ix.2023, leg. N. Meier, T. Spasojevic, A. Viertler (NMBS).

**Remarks.** Here, we report a new *Triclistus* species, represented by a single male specimen. Typically, species descriptions are based on female specimens. Further specimens, particularly females, are required to provide a complete taxonomic diagnosis and confirm the distinctiveness of this species.

#### ﻿Ophioninae

***Enicospilusalbiger* (Kriechbaumer, 1894)**

**Records.** 1 ♂, ZM Eastern Province, South Luangwa nr Mfuwe, xii.2011, A. Gumovsky coll., SAM-HYM-P049484 (SAMC) (Rousse and van Noort 2014b).

***Enicospilusantefurcalis* (Szépligeti, 1908)**

**Records.** 1 ♂, ZM, Mid Luangwa Valley, viii.10 (*Neave*) (NHMUK) (Gauld and Mitchell 1978).

***Enicospilusbiimpressus* (Brullé, 1846)**

**Records.** 1 ♂, ZM, Upper Luangwa River, viii.10 (*Neave*) (NHMUK) (Gauld and Mitchell 1978).

***Enicospiluscapensis* (Thunberg, 1822)**

**Records.** Unspecified, ZM Central Province, Chisamba, Golden Valley Agricultural Research Trust, (14,967,373; 28,097,464, altitude: 1147 m) (Durocher-Granger et al. 2021).

***Enicospilusfenestralis* (Szépligeti, 1906)**

**Records.** 1 ♀, ZM, Luangwa Valley, viii.10 (*Neave*) (NHMUK) (Gauld and Mitchell 1978).

***Enicospilushelvolus* Gauld & Mitchell, 1978**

**Records.** 1 ♂, ZM Northern Province, Mbala, xii.64 (TC) (Gauld and Mitchell 1978).

***Enicospiluslaquaetus* (Enderlein, 1921)**

**Records.** 1 ♀, ZM Lusaka Province, 15 km E Lusaka, Zambia, 22–31.i.1980, R.A. Beaver leg. (NHMUK) (Shimizu et al. 2020).

2 ♀♀, ZM Lusaka Province, 15 km E. of Lusaka, i.1980, R.A. Beaver leg. (NHMUK) (Gauld 1982).

***Enicospilusmauritii* (Saussure, 1892)**

**Records.** 1 ♂, ZM, Upper Luangwa River, vi.1910, leg. S. A. Neave (NHMUK) (Morley 1912).

***Enicospilusnefarius* Gauld & Mitchell, 1978**

**Records.** 1 ♀, ZM Northern Province, Mbala (= Abercorn), xii.64 (TC) (Gauld and Mitchell 1978).

***Enicospilusnops* Gauld & Mitchell, 1978**

**Records.** 2 ♀♀, ZM Northern / Luapula Province, Lake Bangweulu, xi.46 (Steele) (NHMUK) (Gauld and Mitchell 1978).

# ***Enicospilustransvaalensis* Cameron, 1911**

**Records.** 1 ♀, ZM Northern Province, Mbala (= Abercorn), xii.64 (TC) (Gauld and Mitchell 1978).

***Enicospiluswatshami* Gauld, 1982**

**Records.** 1 ♀, ZM Lusaka Province, 15 km E. of Lusaka, i.1980 (R. A. Beaver) (paratype, NHMUK) (Gauld 1982).

***Euryophionlatipennis* (Kirby, 1896)**

**Records.** 1 unspecified, [apex of metasoma lacking], ZM Southern Province, Choma Nansa farm xii.1993, A.J. Gardiner coll., SAM-HYM-P044072 (SAMC) (Rousse and van Noort 2014b)

*Euryophionnigripennis Cameron, 1906

**Records.** 1 ♀, ZM Northern Province, Mpulungu, Kalambo Falls Lodge, sweep net, -8.6241/31.2011, 30.ix.2020, leg. A. Indermaur (NMBS).

***Lepiscelusdistans* (Seyrig, 1935)**

**Records.** 1 ♂, ZM Northern Province, Mbala (= Abercorn), xii.64 (TC) (Gauld and Mitchell 1978).

#### ﻿Orthocentrinae

*Megastylus sp.

**Records.** 1 ♀, ZM Northern Province, Mpulungu, Kalambo River Delta, Malaise trap, 8.5965/31.1844, 21.viii–02.ix.2023, leg. N. Meier, T. Spasojevic, A. Viertler (NMBS).

**Remarks.** This specimen is likely an undescribed species of *Megastylus* common in the Afrotropical region (Augustijn De Ketelaere pers. comm. 2024). However, two of four known species are described only from males, making specimen comparison difficult. Thus, the description of a new species should await a more comprehensive study of Afrotropical *Megastylus*.

#### ﻿Phygadeuontinae

*Paraphylax sp.

**Records.** 1 ♂, ZM Northern Province, Mpulungu, Kalambo Falls Lodge, sweep net, 21.viii–02.ix.2023, leg. N. Meier, T. Spasojevic, A. Viertler (NMBS).

**Remarks.** At this point, the identification of most of the collected phygadeuontines was not possible even to genus level, due to the lack of reference material. Several of the specimens might represent undescribed ichneumonid genera.

#### ﻿Pimplinae

*Theronialurida Tosquinet, 1896

**Records.** 1 ♂, ZM Northern Province, Mpulungu, Kalambo Falls Lodge, sweep net, -8.6241/31.2011, 21.viii–02.ix.2023, leg. N. Meier, T. Spasojevic, A. Viertler (NMBS).

***Xanthopimplaromani* Krieger, 1915**

**Records.** Unspecified, ZM Northern Province, Mbala [Abercorn], 16.vi.1945 (NHMZ) (Benoit 1953b).

2 ♀♀, ZM Northern Province, Chitili stream, sweep net, -8.6390/31.2035, 21.viii–2.ix.2023; leg. N. Meier, T. Spasojevic, A. Viertler (NMBS, LMNH).

***Xanthopimplastemmator* (Thunberg, 1822)**

**Records.** Unspecified, ZM (Midingoyi et al. 2016).

**Remarks.** Introduced in Zambia as a biocontrol agent in the early 2000s.

*Xanthopimpla sp. 1

**Records.** 1 ♀, ZM Northern Province, Mpulungu, Kalambo Falls Lodge, light trap, -8.6241/31.2011, 21.viii–02.ix.2023, leg. N. Meier, T. Spasojevic, A. Viertler (NMBS).

*Xanthopimpla sp. 2

**Records.** 2 ♀♀, ZM Northern Province, Mpulungu, Kalambo Falls Lodge, sweep net, -8.6241/31.2011, 21.viii–02.ix.2023, leg. N. Meier, T. Spasojevic, A. Viertler (NMBS, LMNH).

*Xanthopimpla sp. 3

**Records.** 1 ♀, ZM Northern Province, Mpulungu, Tomo Sakalani, Isanga Bay Lodge, sweep-net, -8.6554/31.1947, 21.viii–2.ix.2023, leg. N. Meier, T. Spasojevic, A. Viertler (NMBS).

**Remarks.** This species belongs to the *X.terebratrix* group of Krieger (1914), but it differs from the known species in several characters and thus might represent a new species.

Several specimens of *Xanthopimpla* could not be identified despite the published identification keys covering almost all the Afrotropical species (Krieger 1914; Seyrig 1932). While some of the species might be new to science, we also found that some key characters used in the keys to distinguish species showed intraspecific variation, which hindered the identification. This suggests that the genus and available keys should be revised.

#### ﻿Tersilochinae

***Diaparsisinterstitialis* Khalaim, 2013**

**Records.** 1 ♀, ZM Copperbelt Province, Kitwe, Chati [Forest Reserve ?], 27.xii.1979, coll. K. Löyttyniemi (MZH) (Khalaim 2019).

***Diaparsismostovskii* Khalaim, 2013**

**Records.** 1 ♀, ZM Western Province, Kalobolelwa, Malaise trap, 11–18.iii.1988, coll. E.G.N. Dijkstra, (RMNH).

1 ♀, Western Province, near Namibian border, Sesheke Town, 950 m, iii–vi.1991, coll. W. Slobbe (RMNH) (Khalaim 2019).

***Diaparsisvoluptuosa* Khalaim, 2013**

**Records.** 2 ♀♀, ZM Copperbelt Province, Kitwe, Chati [Forest Reserve ?], 8.i, 31.iii.1980, coll. K. Löyttyniemi (MZH); 2 ♀, Copperbelt Province, Chati, 9.ii.1980, coll. K. Löyttyniemi (MZH) (Khalaim 2019).

#### ﻿Tryphoninae

***Zambionmonodon* Kasparyan, 1993**

**Records.** 1 ♀, ZM Copperbelt Province, Kitwe, Chati, 27.3.1979, K. Löyttyniemi leg. Label 2: window trap with *Eucalyptus*. Label 3: Holotypus *Zambionmonodon* Kasparyan. Label 4: coll. Dept. Agr. Forest. Zool. Univ. Helsinki, (holotype, MZH). 1 ♂, same data as holotype, except 8.iii.1979, (paratype, MZH). 1 ♀, same data except 15.iii.1979, (paratype, MZH) (Kasparyan 1993).

## ﻿Discussion

### ﻿A checklist with many missing check marks

With only 30 species recorded in the literature, including the major catalogues of the Afrotropical Darwin wasps, the fauna of Zambia is more poorly known than almost any country. Four of these species were previously unrecorded for Zambia in the “Catalogue of world Ichneumonidae” (Yu and Horstmann 1997; Yu et al. 2016) and were added based on careful examination of the original publications of distribution records, highlighting the need for double-proofing information in taxon catalogues. However, the four records as well as approximately half of the total literature records were published during the 20^th^ Century and thus require verification by examining relevant specimens in collections and supplementing them with more recent samples. This is especially true for the records published before Townes`s revision of ichneumonid systematics (Townes 1969, 1970b, 1970a, 1971).

We supplemented the literature-based checklist with species obtained through targeted sampling of Darwin wasps in the field. With only small-scale field work assessment, we increased the species count from the initial 30 species to 44. In addition, we described one species new to science and recorded 17 genera new to Zambia, including *Porizon* which is also recorded for the Afrotropical realm for the first time. This demonstrates that a very large proportion of the Zambian Darwin wasps are still unrecorded or undescribed due to the lack of studies. The finding aligns with a recent species richness estimate for Afrotropical Darwin wasps (Meier et al. 2024), which suggested that only 13–22% of diversity is known in the most studied Afrotropical countries. For example, in Zambia’s neighbouring country Tanzania, between 2,200 and 3,500 species are expected, compared to the ~ 500 recorded species (Meier et al. 2024). Zambia has ¾ of the land mass of Tanzania and lies within the same biogeographic region. This suggests that the current species number of Darwin wasps in Zambia might represent as little as 1.7–2.7% of the actual species richness.

While many faunistic checklists are simply based on literature records, their value can be significantly increased by including specimens available in natural history collections. During our research we discovered a small to medium backlog of unsorted material from Zambia at several European museums of natural history (e.g., NHMUK, LKG) and at the Iziko South African Museum (G. Broad, S. van Noort, M. Schwarz, pers. comm. 2024). This material was not yet included in the present checklist mainly due to two constraints. Firstly, it is not rare that material, including type specimens, from an Afrotropical country is widely distributed across the world. Thus, studying these specimens takes time and substantial funding to visit various collections, which we did not have within the scope of this project. Secondly, the species identification requires a coordinated effort of experts on different subfamilies of Darwin wasps, and many species identifications require generic revisions, which is a long-term process.

### ﻿When genus revisions must forego species identifications

Based on the material collected around the region of Lake Tanganyika, we describe one new Cremastinae species, belonging to the genus *Pristomerus*. This is the only new species of Darwin wasps described based on material from Zambia in the last three years (Di Giovanni and Varga 2021). The designation of the specimens as new species was only possible because of the more recent revisionary works on this genus (Rousse et al. 2011, 2013; Rousse and van Noort 2015). However, for many specimens that we collected in this study, species and sometimes even genus identification was not as straightforward or even possible. A major obstacle to straightforward identification was the lack of identification keys, which are in general rare for the Afrotropical Darwin wasps. Even when keys are present, most of them are rather outdated, with a few exceptions (e.g., Rousse and van Noort 2014b, 2015; Riedel 2016; Rousse et al. 2016; Dal Pos et al. 2024; also available online at WaspWeb (van Noort 2025)). In the absence of revised keys, an examination of original descriptions and usually type material is necessary. However, original descriptions from the 19^th^ Century often just provide a differential diagnosis between species and some of the originally congeneric species were later moved into different genera, making the original descriptions inadequate for differentiating these species from their actual congeners. Unfortunately, also the primary types of a considerable number of species have gone missing, e.g., *Osprynchotusgigas* (Kriechbaumer, 1894). Consequently, within many subfamilies of Darwin wasps, species and often even genus identification is currently not possible in the Afrotropical region. A way forward in species identification involves conducting regional taxonomic revisions of the genera in question, or at least more closely related species, by studying original type material and an adequate number of specimens, and by generating molecular sequences to aid in difficult cases.

### ﻿(Under-) representation of major ecotypes

The Miombo woodlands, where we conducted our fieldwork, represent the most dominant ecoregion in Zambia (Malambo and Syampungani 2008). Although our work took place in this ecoregion, which covers approximately 50% of Zambia’s landmass, we do not consider our sampling to be representative of the actual diversity of Darwin wasps, even within this ecotype. One primary reason is that our sampling was conducted during a brief period in the dry season, while significantly higher activity is reported for many insects during and after the rainy season (Löyttyniemi and Löyttyniemi 1993). Moreover, the previous literature records of Darwin wasps in Zambia are extremely patchy, often based on single specimens and, especially in the older literature, with incomplete locality data. To accurately assess the species diversity of Darwin wasps in Zambia, collecting should be conducted throughout the year (e.g., long-term sampling using Malaise traps) and across various localities, not only covering the central and southern Miombo forests but also extending to other ecotypes. Actually, most of the other major ecoregions of Zambia (Malambo and Syampungani 2008), including the Zambesian flooded grasslands, tropical Cryptosepalum dry forests, Baikiaea woodlands, and Western Zambezian grasslands, have yet to be sampled. Another factor limiting the representativeness of our sampling, especially when it comes to the species endemic to Zambia, is the proximity of our sampling area to the Tanzanian border, where considerable overlap with the species assemblage of Tanzania is likely.

### ﻿Outlook

In summary, extensive and collaborative collection efforts across all Zambian ecotypes, involving both local research institutions and international taxonomic specialists, are needed to grasp the species richness of Darwin wasps in Zambia. This observation likely holds true not only for Zambia and Darwin wasps but also for most of the Afrotropical region and insect groups (e.g., Azevedo et al. 2015; van Noort et al. 2015; Salden and Peters 2023). We here present a preliminary step towards developing a comprehensive checklist of Darwin wasps for the country. Despite conducting fieldwork at the end of the dry season and near the border with Tanzania, a country with better-studied Darwin wasp diversity in the Afrotropical region, our findings suggest significant opportunities for further discovery.

Building on the insights gained from this initial study, we suggest several improvements for future fieldwork. These include extending the use of Malaise traps over longer periods and fostering collaborations with local universities and students. Additionally, the fieldwork should be conducted during or closer to the rainy season to capture a broader range of species. Another important next step is to examine several natural history collections containing already sampled Zambian insects. Studying these collections might yield many additional species records and new species descriptions given the extent of this so far mostly unidentified material.

Finally, recent global awareness of biodiversity loss has pushed initiatives aimed at documenting and monitoring biodiversity in Afrotropical countries, including Zambia. These efforts have highlighted the region’s immense yet understudied biological richness. Major collection initiatives, such as the expeditions of the African Natural History Research Trust focusing on Lepidoptera, highlight the value of large-scale, collaborative fieldwork. Similar initiatives targeting hymenopteran diversity, such as the extensive surveys of the Afrotropical Hymenoptera Initiative conducted over the last 33 years by Simon van Noort of the Iziko South African Museum in Cape Town (van Noort 2025) could yield insights and help increase understanding of their diversity in the Afrotropical region, while strengthening the local capacity for taxonomic work through education and enlargement of the local natural history collections.

## Supplementary Material

XML Treatment for Pristomerus roussei

## ﻿Additional information

### Author ORCIDs

Noah Meier https://orcid.org/0000-0003-2139-4339

Alexandra Viertler https://orcid.org/0000-0001-9729-5439

Meekness Kapaale https://orcid.org/0000-0001-9745-9835

Cyprian Katongo https://orcid.org/0000-0002-5276-3216

Tamara Spasojevic https://orcid.org/0000-0001-5301-5722

### Data availability

All of the data that support the findings of this study are available in the main text.
